# A rapid pyrosequencing assay for the molecular detection of influenza viruses with reduced baloxavir susceptibility due to PA/I38X amino acid substitutions

**DOI:** 10.1111/irv.12725

**Published:** 2020-02-11

**Authors:** Paulina Koszalka, Rubaiyea Farrukee, Edin Mifsud, Dhanasekaran Vijaykrishna, Aeron C. Hurt

**Affiliations:** ^1^ WHO Collaborating Centre for Reference and Research on Influenza VIDRL Peter Doherty Institute for Infection and Immunity Melbourne VIC Australia; ^2^ Department of Microbiology Biomedicine Discovery Institute Monash University Melbourne VIC Australia; ^3^ Department of Microbiology and Immunology University of Melbourne Parkville VIC Australia

**Keywords:** antiviral resistance, baloxavir marboxil, I38T, influenza viruses, pyrosequencing

## Abstract

Baloxavir marboxil is a novel endonuclease inhibitor licensed for treatment of otherwise healthy or high‐risk individuals infected with influenza. Viruses with reduced baloxavir susceptibility due to amino acid substitutions at residue 38 of the PA have been detected in some individuals following treatment. Here, we describe a genotypic pyrosequencing method that can be used to rapidly screen circulating influenza A and B viruses for substitutions in the PA/I38 codon and to quantify mixed viral populations. This method is suitable for surveillance of baloxavir susceptibility and to analyse samples from hospitalised patients undergoing baloxavir treatment to aid in clinical decision making.

## 1 INTRODUCTION

Baloxavir marboxil is a small molecule inhibitor of the endonuclease region in the polymerase acidic (PA) protein of influenza viruses and was recently licensed for treatment of influenza in otherwise healthy and high‐risk individuals in Japan and the United States.^1^ Analysis of viruses obtained from patients following treatment revealed that amino acid substitutions at residue 38 of the PA protein (I38T, I38M or I38F, referred to as PA/I38X) confer 10‐fold to 68‐fold reductions in baloxavir susceptibility in vitro.^2^, ^3^ These substitutions are detected at variable frequencies in baloxavir‐treated patients, with the highest rates in adolescents infected with A(H3N2) viruses, where PA/I38X substitutions were identified in 23.4% of patients.^2^ To date, PA/I38T is the most commonly detected substitution and is associated with the largest reduction in baloxavir susceptibility (50‐fold to 68‐fold compared with wild‐type virus).^2^, ^3^

In the 2018/19 influenza season, over six million people were treated with baloxavir in Japan and PA/I38X substitutions were reported in 6/335 (1.5%) of A(H1N1pdm09) viruses, 34/356 (9.6%) of A(H3N2) and 0/42 of influenza B viruses by The National Institute of Infectious Diseases (NIID, Japan). Viruses that contain PA/I38T substitutions were also detected in four patients who had not been treated with baloxavir, suggesting that variant viruses had transmitted between people.^4^ Given the current rates of PA/I38X variants obtained from baloxavir‐treated patients and the potential transmissibility of these viruses, surveillance is important to monitor for the emergence of PA/I38X variants in the community. Importantly, rapid detection of viruses with reduced antiviral susceptibility in hospitalised patients can aid clinicians in selecting appropriate antiviral drugs and improve patient management.

Point‐of‐care tests are available for the rapid detection of influenza infection, however, these tests do not have the capacity to provide information on the presence of specific amino acid substitutions. Therefore, laboratory assays are utilised to determine antiviral susceptibility. Phenotypic assays that directly measure baloxavir susceptibility have been developed 5, 6, 7; however these assays typically require cultured isolates, are slow (3‐5 days) and relatively low throughput. Consequently, rapid genotypic assays which can be performed directly on clinical specimens are required. Pyrosequencing has been previously utilised to detect amino acid substitutions that are known to confer reduced susceptibility to M2 ion channel inhibitors and neuraminidase inhibitors.^8^ Here, we outline a pyrosequencing method for the detection of PA/I38X variants in A(H3N2), A(H1N1pdm09) and influenza B viruses and report on the accuracy of sequence analysis and estimated mixture proportions.

Full‐length PA nucleotide sequences for all circulating influenza subtypes/types submitted to the Global Initiative on Sharing All Influenza Data (GISAID) database from 2009 to 2018 were downloaded. For each virus type/subtype, nucleotide sequences were aligned using MAFFT and primer sets were designed such that they bound to regions of high similarity (>90% conservation of sequences)^9^ (Table 1). RNA was extracted using the QIAamp Viral RNA kit (Qiagen) according to the manufacturer's protocol, and RT‐PCR was conducted using the MyTaq One‐Step RT‐PCR kit (Bioline) and standard thermocycling conditions.^10^ The PyroMark vacuum prep workstation, PyroMark ID Q96 and PyroMark gold reagents (Qiagen) were used as previously described.^11^

**Table 1:** RT‐PCR and pyrosequencing primer sequences

Influenza type/subtype	RT‐PCR forward	RT‐PCR reverse	Sequencing
A(H1N1)pdm09	Biotin‐CAATCCAATGATCGTCGAGC	GGTGCTTCAATAGTGCATTTGG	CAAACTTCCAAATGTGTGCA
A(H3N2)	Biotin‐TTGTCGAACTTGCAGAAAAGGC	GCCATTGTTCTGTCTCTCCCCT	CATACCTCCAAGTGAGTGCA
Influenza B	Biotin‐ATACAAAAGGCCAAAAACACAATG	GTTCTTTCCCTTGTCCTTCTAATGC	GCAAACCTCTAGATGGACRCA

Note

All primers in 5′‐3′ orientation. Sequencing primers are in the reverse complement.

Pyrosequencing assays require a standard RT‐PCR reaction in conjunction with specific primers designed for amplification of the PA segment that encodes codon 38, specifically, nucleotide 38‐260 (A(H3N2)), 27‐223 (A(H1N1pdm09)) or 40‐211 (Influenza B). The forward primer is biotinylated to enable binding to streptavidin beads later in the assay.

**Figure 1:**
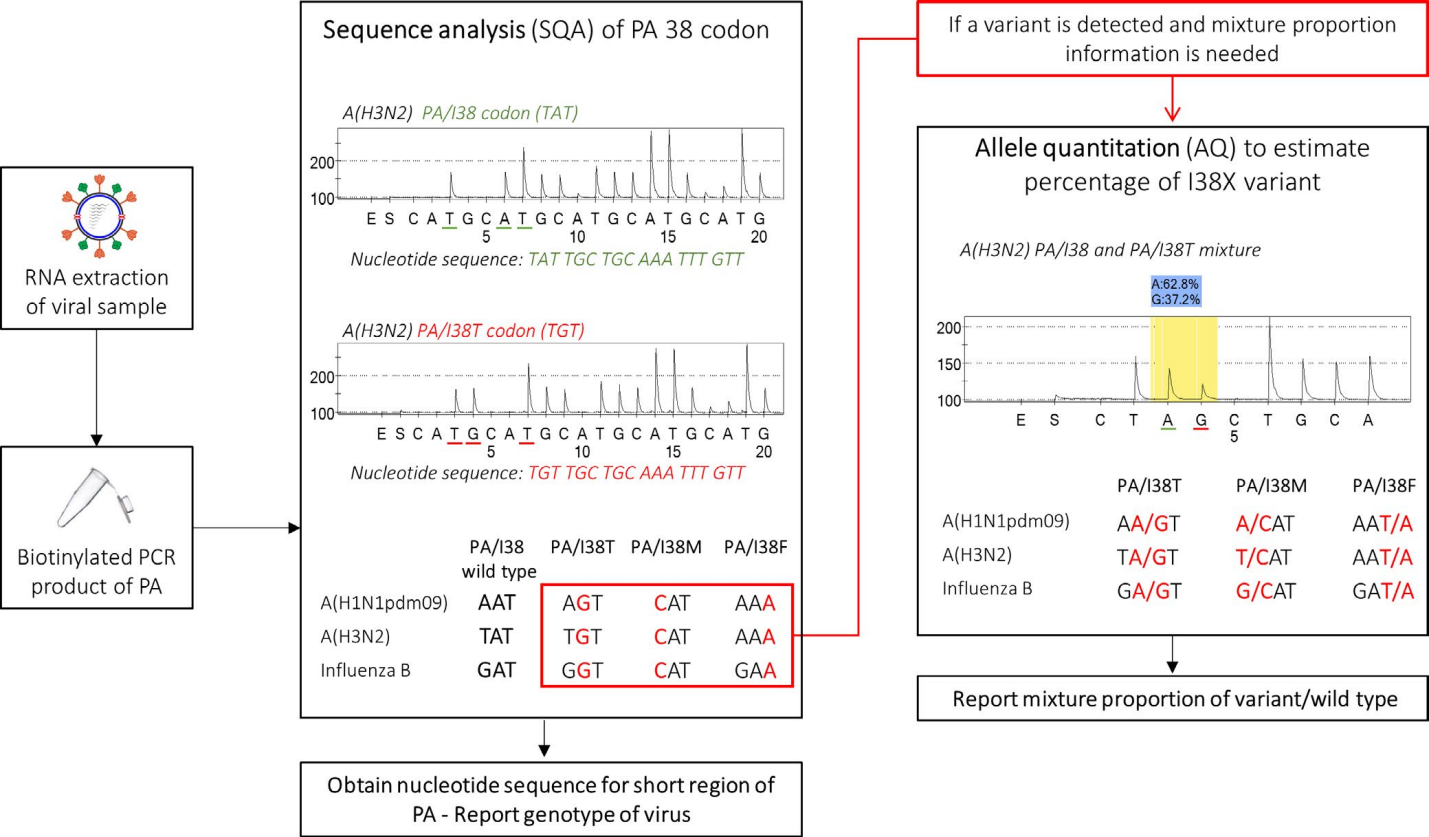
Workflow for the identification of PA/I38X variants using pyrosequencing. The PyromarkID system and Pyromark Gold reagents in conjunction with the primers in Table 1 are used for the identification of PA/I38X variants in influenza samples. RNA is extracted from influenza virus samples and RT‐PCR with biotin‐tagged primers is used to amplify an approximately 100 base pair segment that encompasses the region of interest in the PA protein. Using the sequence analysis mode of the Pyromark ID, a nucleotide sequence surrounding codon 38 is obtained. The sequence analysis panel of the diagram depicts PA/I38 and PA/I38T pyrogram, with the y‐axis as a luminescence measure and the x‐axis showing the addition of enzyme (E), substrate (S) and nucleotides A, T, G, C. The peak height is representative of nucleotide addition. If a variant is identified, the sample may be further characterised for mixture proportion using the allele quantitation mode of the Pyromark ID. Using half volumes of the initial biotinylated PCR product allows the same PCR product to be used for both sequence analysis and allele quantitation. Codons are depicted as the reverse complement as the biotin tag for the pyrosequencing primers will sequence in the reverse direction

The workflow for identifying PA/I38X variants is depicted in Figure 1, where the sequence of the PA/I38 codon is determined using the “sequence analysis” (SQA) mode of the PyroMarkID Q96. A biotinylated PCR product will yield a pyrogram and a nucleotide sequence for a short region (approximately 15‐30 base pairs) that encompasses the single nucleotide polymorphism (SNP) of interest. As a result, the presence of an amino acid substitution can be identified. As biotin is tagged on the forward primer of the RT‐PCR reaction, the codons depicted in Figure 1 are in the reverse complement. It is also important to note that the codon sequence for the A(H1N1pdm09) wild‐type PA/I38 was TAT (ATA, forward direction) prior to 2015 but has since changed to AAT (ATT, forward direction). Once the nucleotide sequence for a virus is obtained and an amino acid substitution is detected, the relative proportion of the wild type and variant mixture proportion can be assessed using the “Allele Quantitation (AQ)” mode. The AQ mode will estimate the proportion of the two nucleotides of interest based on the relative height of the pyrogram peak. This additional step is only necessary if detailed mixture analysis is required (Figure 1).

The specificity of the pyrosequencing primers was tested with 60 representative cultured virus isolates (including A(H1N1)pdm09, A(H3N2), B/Victoria, B/Yamagata) collected as part of the WHO Global Influenza Surveillance and Response System from various geographic locations between 2011 and 2018. All viruses were successfully amplified by the primers, and SQA analysis indicated that all viruses contained the PA/I38 wild‐type codon. As part of routine surveillance, 71 influenza virus isolates circulating in 2019 were tested, and all were successfully amplified and shown to contain a wild‐type codon at PA/I38.

To evaluate the sensitivity and limit of detection of the pyrosequencing assay, three virus isolates A/Tasmania/501/2018 (H1N1pdm09), A/Sydney/21/2019 (H3N2) and B/Perth/14/2018 (B/Yamagata lineage) were titrated in 10‐fold dilutions from 10^5^ to 10^1^ RNA copy number/mL. PyroMarkID SQA software will provide a “pass,” “check” or “fail” quality score for each sequence. SQA analysis was successfully performed with a “pass” quality check on all virus types/subtypes until 10^3^ RNA copies/mL. A “check” quality score was obtained at 10^2^ RNA copies/mL for all virus types; however, a nucleotide sequence was still generated by the PyroMark ID software. For sequences generated with the “check” quality score, pyrograms need to be visually checked for markers of poor quality sequence such as wide peaks, spurious peaks, initial baseline drift or overall low signal. At 10^1^ RNA copies/mL, the pyrosequencing method we have described cannot obtain a sequence for all influenza virus types and a “fail” quality score was obtained.

Mixed viral populations of wild type (WT) and variant viruses can occur in patients undergoing antiviral treatment.^12^ The accuracy of the pyrosequencing assay to detect PA/I38X:WT mixtures was tested using DNA plasmids containing the PA segment from A/Perth/261/2009 (H1N1pdm09), A/Perth/16/2009 (H3N2) and B/Yamanashi/166/1998 (B Yamagata lineage) with either a WT or variant codon that was generated through site‐directed mutagenesis. Plasmids were quantified using the Quant‐iT™ PicoGreen™ assay and tested as unmixed pure stocks or mixtures of WT/variant codon plasmids. Samples were quantified using the AQ mode to determine the percentage of variant (Table 2). For all viruses, the assay was accurate in estimating PA/I38T:WT and PA/I38M:WT mixtures, for example the 50% A/Perth/261/2009 PA/i38T mixture was detected as 48.8%. Poor accuracy was detected for PA/I38F:WT mixtures, where the B/Yamanashi/166/1998 PA/I38F 50% mixture was detected as 35.8%. The PyroMark ID software does not always have the ability to accurately quantify the chromatogram peak height of homopolymers, this may cause the poor accuracy shown for the influenza A PA/I38F codon for which the nucelotide sequence is AAA (reverse complement).^13^ Mixture proportions were generated for the influenza B PA/I38F:WT but the estimate was 10%‐15% lower than expected. The error associated with estimating pure populations of PA/I38 or PA/I38X is such that percentages that are <5% or >95% cannot be accurately quantified (Table 2).

**Table 2:** Accuracy of mixture estimates by the AQ pyrosequencing assay across different influenza virus types/subtype and PA/I38 substitutions

	Expected variant (%)	Percentage of variant detected by pyrosequencing from a mixed population of WT and indicated PA/I38X variant		
		PA/I38T	PA/I38M	PA/I38F
A(H1N1pdm09)	0	2.4 ± 0.3	0 ± 0	ND
A/Perth/261/2009	25	23.8 ± 0.4	23.3 ± 0.3	ND
	50	48.8 ± 0.5	47.8 ± 0.4	ND
	75	72.7 ± 0.6	72.1 ± 0.3	ND
	100	98.3 ± 0.2	100 ± 0	ND
A(H3N2)	0	0.5 ± 0.7	1.3 ± 0.1	ND
A/Perth/16/2009	25	27.3 ± 0.5	27.2 ± 0.5	ND
	50	52.6 ± 0.3	51.9 ± 0.6	ND
	75	73.9 ± 0.4	74.3 ± 0.1	ND
	100	98.3 ± 0.4	99.3 ± 0.5	ND
B/Yamagata lineage	0	1.2 ± 1.7	0.4 ± 0.6	0 ± 0
B/Yamanashi/166/1998	25	31.7 ± 0.4	25.8 ± 0.5	9.5 ± 1.3
	50	49.7 ± 0.4	47.5 ± 0.5	35.8 ± 5.5
	75	71.6 ± 0.2	72.1 ± 0.5	63.7 ± 3.2
	100	98.4 ± 1.7	100 ± 0	100 ± 0

Note

Not determined (ND) ‐ AQ analysis could not be performed by PyroMarkID software.

Variant percentages are the average ± standard deviation of three experiments.

To evaluate the accuracy of the mixture proportion estimates at low RNA levels, a PA/I38T:WT virus mixture (reverse genetics derived A/Perth/261/2009) was prepared and titrated 10‐fold from 10^7^ to 10^1^ RNA copy number/mL. AQ analysis of these samples showed that mixture proportions remained consistent until 10^3^ copy number/mL (Table 3).

**Table 3:** Limit of accuracy for AQ analysis

RNA copy number/mL	Proportion of PA/I38T (%)	PyroMark ID quality score
10^7^	36.6 ± 0.8	Pass
10^6^	36.6 ± 0.9	Pass
10^5^	38.0 ± 2.5	Pass
10^4^	38.6 ± 0.4	Pass
10^3^	39.3 ± 0.2	Pass
10^2^	59.2 ± 2.0	Check
10^1^	27.2 ± 25.7	Fail

Note

PA/I38T proportions are the average ± standard deviation of three experiments.

The main analysis to determine the accuracy of the assay was conducted with varying mixtures of plasmids rather than viruses to improve the accuracy of mixture preparation. However, when equivalent mixtures of live viruses have been prepared, the assay has performed similarly (data not shown). Laboratories that are establishing this assay may wish to validate the primers and assay on clinical material from patients.

Pyrosequencing has several benefits for the detection of SNPs to infer antiviral susceptibility. It is rapid, high‐throughput, easy to analyse and can provide the quantification of wild type and variant mixtures. The relatively high sensitivity of our assay also allows for detection of PA/I38X variants and mixture estimates in clinical specimens with low RNA copy numbers and does not require a cultured isolate. This allows for a turn‐around time of <4 hours, meaning antiviral treatment of hospitalised patients with influenza can be altered in a clinically relevant window if a variant is detected. This is important for patients who are treated with antivirals for prolonged periods (such as immunocompromised patients) as continual viral shedding under drug selection pressure has been shown to select variants with reduced drug susceptibility.^12^

Amino acid substitutions known to confer reduced susceptibility to antiviral compounds can also be determined with other genotypic methods such as qPCR, Sanger sequencing and next‐generation sequencing (NGS). Although qPCR is a high‐throughput method that is widely established in laboratories and is relatively inexpensive, the variety of PA/I38X substitutions already observed in clinical specimen means that separate assays would be required for each potential substitution and the sequence of the PCR products is not usually obtained so novel changes at PA/I38X would not be identified.^14^ Sanger sequencing is of moderate cost and is accessible but requires several days to generate sequences and is relatively inaccurate in assessing viral mixtures.^14^ NGS has higher accuracy for viral mixture quantification but requires extensive sample preparation and data analysis.

Pyrosequencing is frequently performed for identifying reduced susceptibility to the neuraminidase inhibitors (NA/H275Y) and the M2 ion channel inhibitors (M2/S31N), although the equipment is not as commonly available in laboratories as real‐time PCR thermocyclers.^15^ The pyrosequencing method described in this study can be used for high‐throughput screening of circulating influenza viruses for PA/I38X amino acid substitutions, and further analysis for mixtures of PA/I38X and wild‐type sequences provides information on the emergence of variant viruses in patients receiving treatment.
